# Supplementary material for: Noble Gas—Silicon Cations: Theoretical Insights into the Nature of the Bond
Source: Molecules. 2022 Jul 19;27(14):4592. doi: 10.3390/molecules27144592 (PMC9323317; doi:10.3390/molecules27144592)
Supplement: Supplementary file 1 [file molecules-27-04592-s001.zip › molecules-1814532-supplementary.pdf]

Noble Gas – Silicon Cations: Theoretical Insights into the Nature of the Bond

Stefano Borocci,[a,b] Felice Grandinetti,*[,a,b] and Nico Sanna[a,c]

to be published in *Molecules*

---

[a] *Prof. S. Borocci, Prof. F. Grandinetti, Prof. N. Sanna*

Dipartimento per la Innovazione nei sistemi Biologici, Agroalimentari e Forestali (DIBAF), Università della Tuscia, L.go dell'Università, s.n.c., 01100 Viterbo (Italy)

E-mail: fgrandi@unitus.it

orcid.org/0000-0001-7217-9490

[b] *Prof. S. Borocci, Prof. F. Grandinetti*

Istituto per i Sistemi Biologici del CNR, Via Salaria, Km 29.500, 00015, Monterotondo, RM (Italy)

[c] *Prof. N. Sanna*

Istituto per la Scienza e Tecnologia dei Plasmi (ISTP) del CNR,Via Amendola, 122/D, 70126 Bari (Italy)

Supplementary Information

Data S1. MP2/aug-cc-pVTZ optimized cartesian coordinates (Å) of the Ng-Si cations.

$HeSiH_3^+$

He	0.00000000	0.00000000	0.00000000
Si	0.00000000	0.00000000	2.12924015
H	1.46007416	0.00000000	2.15762808
H	-0.73003708	1.26446131	2.15762808
H	-0.73003708	-1.26446131	2.15762808

$NeSiH_3^+$

Ne	0.00000000	0.00000000	0.00000000
Si	0.00000000	0.00000000	2.32988195
H	1.45960979	0.00000000	2.37075790
H	-0.72980489	1.26405916	2.37075790
H	-0.72980489	-1.26405916	2.37075790

$ArSiH_3^+$

Ar	0.00000000	0.00000000	0.00000000
Si	0.00000000	0.00000000	2.40634108
H	1.45406685	0.00000000	2.53958491
H	-0.72703342	1.25925883	2.53958491
H	-0.72703342	-1.25925883	2.53958491

$KrSiH_3^+$

Kr	0.00000000	0.00000000	0.00000000
Si	0.00000000	0.00000000	2.51405822
H	1.45171616	0.00000000	2.67791856
H	-0.72585808	1.25722307	2.67791856
H	-0.72585808	-1.25722307	2.67791856

$XeSiH_3^+$

Xe	0.00000000	0.00000000	0.00000000
Si	0.00000000	0.00000000	2.66440791
H	1.44837808	0.00000000	2.86530110
H	-0.72418904	1.25433221	2.86530110
H	-0.72418904	-1.25433221	2.86530110

$RnSiH_3^+$

Rn	0.00000000	0.00000000	0.00000000
Si	0.00000000	0.00000000	2.73640309
H	1.44683491	0.00000000	2.95318289
H	-0.72341746	1.25299579	2.95318289
H	-0.72341746	-1.25299579	2.95318289

$HeSiF_3^+$

He	0.00000000	0.00000000	0.00000000
Si	0.00000000	0.00000000	2.05986449
F	1.53026480	0.00000000	2.11153304
F	-0.76513240	1.32524819	2.11153304
F	-0.76513240	-1.32524819	2.11153304

$NeSiF_3^+$

Ne	0.00000000	0.00000000	0.00000000
Si	0.00000000	0.00000000	2.18824200
F	1.53025327	0.00000000	2.27053443
F	-0.76512663	1.32523820	2.27053443
F	-0.76512663	-1.32523820	2.27053443

$ArSiF_3^+$

Ar	0.00000000	0.00000000	0.00000000
Si	0.00000000	0.00000000	2.29713447
F	1.52486472	0.00000000	2.51697995
F	-0.76243236	1.32057159	2.51697995
F	-0.76243236	-1.32057159	2.51697995

$KrSiF_3^+$

Kr	0.00000000	0.00000000	0.00000000
Si	0.00000000	0.00000000	2.41538228
F	1.52207916	0.00000000	2.67626544
F	-0.76103958	1.31815922	2.67626544
F	-0.76103958	-1.31815922	2.67626544

$XeSiF_3^+$

Xe	0.00000000	0.00000000	0.00000000
Si	0.00000000	0.00000000	2.57601399
F	1.51810503	0.00000000	2.88551776
F	-0.75905252	1.31471752	2.88551776
F	-0.75905252	-1.31471752	2.88551776

$RnSiF_3^+$

Rn	0.00000000	0.00000000	0.00000000
Si	0.00000000	0.00000000	2.65219476
F	1.51640125	0.00000000	2.98247877
F	-0.75820063	1.31324201	2.98247877
F	-0.75820063	-1.31324201	2.98247877

$HeSiCl_3^+$

He	0.00000000	0.00000000	0.00000000
Si	0.00000000	0.00000000	2.98596866
Cl	1.95711262	0.00000000	2.99117047
Cl	-0.97855631	1.69490925	2.99117047
Cl	-0.97855631	-1.69490925	2.99117047

$NeSiCl_3^+$

Ne	0.00000000	0.00000000	0.00000000
Si	0.00000000	0.00000000	2.91554061
Cl	1.95729197	0.00000000	2.93207681
Cl	-0.97864598	1.69506457	2.93207681
Cl	-0.97864598	-1.69506457	2.93207681

$ArSiCl_3^+$

Ar	0.00000000	0.00000000	0.00000000
Si	0.00000000	0.00000000	2.69040654
Cl	1.95786108	0.00000000	2.84789956
Cl	-0.97893054	1.69555743	2.84789956
Cl	-0.97893054	-1.69555743	2.84789956

$KrSiCl_3^+$

Kr	0.00000000	0.00000000	0.00000000
Si	0.00000000	0.00000000	2.66961586
Cl	1.95551282	0.00000000	2.91811496
Cl	-0.97775641	1.69352378	2.91811496
Cl	-0.97775641	-1.69352378	2.91811496

$XeSiCl_3^+$

Xe	0.00000000	0.00000000	0.00000000
Si	0.00000000	0.00000000	2.74597389
Cl	1.95127808	0.00000000	3.08320187
Cl	-0.97563904	1.68985638	3.08320187
Cl	-0.97563904	-1.68985638	3.08320187

$RnSiCl_3^+$

Rn	0.00000000	0.00000000	0.00000000
Si	0.00000000	0.00000000	2.79282755
Cl	1.94940089	0.00000000	3.16481097
Cl	-0.97470044	1.68823069	3.16481097
Cl	-0.97470044	-1.68823069	3.16481097

$Ar_2SiH_3^+$

H	0.00000000	0.00000000	0.00000000
Si	0.00000000	0.00000000	1.45830103
Ar	2.56984552	0.00000000	1.45830103
Ar	-2.56984552	0.00000000	1.45830103
H	0.00000000	1.26292574	2.18745155
H	0.00000000	-1.26292574	2.18745155

$Ar_2SiF_3^+$

F	0.00000000	0.00000000	0.00000000
Si	0.00000000	0.00000000	1.54439554
Ar	2.49103590	0.00000000	1.54439554
Ar	-2.49103590	0.00000000	1.54439554
F	0.00000000	1.33748577	2.31659331
F	0.00000000	-1.33748577	2.31659331

$Ar_2SiCl_3^+$

Cl	0.00000000	0.00000000	0.00000000
Si	0.00000000	0.00000000	1.96315153
Ar	2.96064596	0.00000000	1.96315153
Ar	-2.96064596	0.00000000	1.96315153
Cl	0.00000000	1.70013910	2.94472730
Cl	0.00000000	-1.70013910	2.94472730

$HeSiF_2^{2+}$

He	0.00000000	0.00000000	0.00000000
Si	0.00000000	0.00000000	1.72079363
F	1.48502704	0.00000000	1.97266001
F	-1.48502704	0.00000000	1.97266001

$NeSiF_2^{2+}$

Ne	0.00000000	0.00000000	0.00000000
Si	0.00000000	0.00000000	1.88787057
F	1.47278802	0.00000000	2.21105249
F	-1.47278802	0.00000000	2.21105249

$ArSiF_2^{2+}$

Ar	0.00000000	0.00000000	0.00000000
Si	0.00000000	0.00000000	2.11152821
F	1.42239965	0.00000000	2.62970922
F	-1.42239965	0.00000000	2.62970922

$KrSiF_2^{2+}$

Kr	0.00000000	0.00000000	0.00000000
Si	0.00000000	0.00000000	2.24666591
F	1.40348131	0.00000000	2.82355207
F	-1.40348131	0.00000000	2.82355207

$XeSiF_2^{2+}$

Xe	0.00000000	0.00000000	0.00000000
Si	0.00000000	0.00000000	2.42804609
F	1.38125931	0.00000000	3.06918659
F	-1.38125931	0.00000000	3.06918659

$RnSiF_2^{2+}$

Rn	0.00000000	0.00000000	0.00000000
Si	0.00000000	0.00000000	2.51774213
F	1.37171708	0.00000000	3.18580290
F	-1.37171708	0.00000000	3.18580290

$FKrSiF_2^{+}$

Kr	0.00000000	0.00000000	0.00000000
Si	0.00000000	0.00000000	2.47668318
F	1.31084818	0.00000000	3.30975101
F	-1.31084818	0.00000000	3.30975101
F	0.00000000	0.00000000	-1.92648414

$FXeSiF_2^{+}$

Xe	0.00000000	0.00000000	0.00000000
Si	0.00000000	0.00000000	2.63044819
F	1.29886176	0.00000000	3.48814902
F	-1.29886176	0.00000000	3.48814902
F	0.00000000	0.00000000	-1.98253314

$FRnSiF_2^{+}$

Rn	0.00000000	0.00000000	0.00000000
Si	0.00000000	0.00000000	2.73457920
F	1.29349491	0.00000000	3.60456496
F	-1.29349491	0.00000000	3.60456496
F	0.00000000	0.00000000	-2.05362115

Table S1. MP2/aug-cc-pVTZ cartesian coordinates (Å) and AIM indices of the BCP located on the Ng-Si bonds of the Ng-Si cations. The origin is at Ng except for $Ar_2SiX_3^+$ (origin at X).

	Bond	X	Y	Z	ρ^a	$\nabla^2(\rho)^b$	H^c
$HeSiH_3^+$	He-Si	0.0	0.0	1.0826	0.0163	0.0581	-0.0003
$NeSiH_3^+$	Ne-Si	0.0	0.0	1.2907	0.0176	0.0553	-0.0017
$ArSiH_3^+$	Ar-Si	0.0	0.0	1.5272	0.0359	0.0792	-0.0109
$KrSiH_3^+$	Kr-Si	0.0	0.0	1.6368	0.0402	0.0569	-0.0156
$XeSiH_3^+$	Xe-Si	0.0	0.0	1.7716	0.0447	0.0153	-0.0214
$RnSiH_3^+$	Rn-Si	0.0	0.0	1.8204	0.0460	-0.0092	-0.0233
$HeSiF_3^+$	He-Si	0.0	0.0	1.0809	0.0183	0.0651	-0.0007
$NeSiF_3^+$	Ne-Si	0.0	0.0	1.25995	0.0230	0.0790	-0.0028
$ArSiF_3^+$	Ar-Si	0.0	0.0	1.4773	0.0466	0.1237	-0.0157
$KrSiF_3^+$	Kr-Si	0.0	0.0	1.5916	0.0516	0.0791	-0.0232
$XeSiF_3^+$	Xe-Si	0.0	0.0	1.7361	0.0571	0.0093	-0.0326
$RnSiF_3^+$	Rn-Si	0.0	0.0	1.7920	0.0589	-0.0320	-0.0362
$HeSiCl_3^+$	He-Si	0.0	0.0	1.29325	0.0036	0.0153	0.00096
$NeSiCl_3^+$	Ne-Si	0.0	0.0	1.4241	0.0071	0.0239	0.0005
$ArSiCl_3^+$	Ar-Si	0.0	0.0	1.5917	0.0238	0.0293	-0.0047
$KrSiCl_3^+$	Kr-Si	0.0	0.0	1.6748	0.0337	0.0119	-0.0122
$XeSiCl_3^+$	Xe-Si	0.0	0.0	1.7733	0.0430	-0.0187	-0.0200
$RnSiCl_3^+$	Rn-Si	0.0	0.0	1.7917	0.0464	-0.0348	-0.0220
$Ar_2SiH_3^+$	Ar-Si	0.9922	0.0	1.4583	0.0265	0.0449	-0.0064
$Ar_2SiF_3^+$	Ar-Si	0.9175	0.0	1.5444	0.0314	0.0512	-0.0102
$Ar_2SiCl_3^+$	Ar-Si	1.3022	0.0	1.9632	0.0144	0.0291	-0.00056
$HeSiF_2^{2+}$	He-Si	0.0	0.0	0.9290	0.0431	0.2378	-0.0024
$NeSiF_2^{2+}$	Ne-Si	0.0	0.0	1.1094	0.0500	0.2926	-0.0023
$ArSiF_2^{2+}$	Ar-Si	0.0	0.0	1.3569	0.0784	0.2228	-0.0362
$KrSiF_2^{2+}$	Kr-Si	0.0	0.0	1.4773	0.0821	0.1102	-0.0476
$XeSiF_2^{2+}$	Xe-Si	0.0	0.0	1.6259	0.0851	-0.0325	-0.0588
$RnSiF_2^{2+}$	Rn-Si	0.0	0.0	1.6649	0.0848	-0.1190	-0.0607
$FKrSiF_2^+$	Kr-F	0.0	0.0	-1.0141	0.1300	0.2526	-0.0552
	Kr-Si	0.0	0.0	1.3361	0.0754	-0.0297	-0.0297
$FXeSiF_2^+$	Xe-F	0.0	0.0	-1.0223	0.1290	0.2395	-0.0680
	Xe-Si	0.0	0.0	1.3720	0.0716	-0.0143	-0.0255
$FRnSiF_2^+$	Rn-F	0.0	0.0	-1.0904	0.1190	0.3299	-0.04995
	Rn-Si	0.0	0.0	1.4001	0.0633	0.0096	-0.0204

[a] Electron density ($e\ a_0^{-3}$). [b] Laplacian of the electron density ($e\ a_0^{-5}$). [c] Energy density (*hartree* a_0^{-3}).

Table S2. CCSD/aug-cc-pVTZ T1 diagnostics of the Ng-Si cations.

	T1		T1
$HeSiH_3^+$	0.0079	$KrSiCl_3^+$	0.0099
$NeSiH_3^+$	0.0089	$XeSiCl_3^+$	0.0100
$ArSiH_3^+$	0.0074	$RnSiCl_3^+$	0.0103
$KrSiH_3^+$	0.0082	$Ar_2SiH_3^+$	0.0067
$XeSiH_3^+$	0.0094	$Ar_2SiF_3^+$	0.0108
$RnSiH_3^+$	0.0105	$Ar_2SiCl_3^+$	0.0092
$HeSiF_3^+$	0.0133	$HeSiF_2^{2+}$	0.0170
$NeSiF_3^+$	0.0126	$NeSiF_2^{2+}$	0.0154
$ArSiF_3^+$	0.0119	$ArSiF_2^{2+}$	0.0144
$KrSiF_3^+$	0.0120	$KrSiF_2^{2+}$	0.0145
$XeSiF_3^+$	0.0122	$XeSiF_2^{2+}$	0.0148
$RnSiF_3^+$	0.0124	$RnSiF_2^{2+}$	0.0152
$HeSiCl_3^+$	0.0111	$FKrSiF_2^+$	0.0202
$NeSiCl_3^+$	0.0107	$FXeSiF_2^+$	0.0184
$ArSiCl_3^+$	0.0100	$FRnSiF_2^+$	0.0181
